# Prior Precision, Prior Accuracy, and the Estimation of Disease Prevalence Using Imperfect Diagnostic Tests

**DOI:** 10.3389/fvets.2018.00083

**Published:** 2018-05-11

**Authors:** Jenni L. McDonald, Dave James Hodgson

**Affiliations:** Centre for Ecology and Conservation, College of Life and Environmental Sciences, University of Exeter, Penryn, United Kingdom

**Keywords:** diagnostics, Bayesian inference, sensitivity, specificity, prevalence, bovine tuberculosis, accuracy, precision

## Abstract

Estimates of disease prevalence in any host population are complicated by uncertainty in the outcome of diagnostic tests on individuals. In the absence of gold standard diagnostics (tests that give neither false positives nor false negatives), Bayesian latent class inference can be applied to batteries of diagnostic tests, providing posterior estimates of the sensitivity and specificity of each test, alongside posterior estimates of disease prevalence. Here we explore the influence of precision and accuracy of prior information on the precision and accuracy of posterior estimates of these key parameters. Our simulations use three diagnostic tests, yielding eight possible diagnostic outcomes per individual. Seven degrees of freedom allow the estimation of seven parameters: sensitivity and specificity of each test, and disease prevalence. We show that prior precision begets posterior precision but only when priors are accurate. We also show that analyses without gold standard can use imprecise priors as long as they are initialised with accuracy. Imprecise priors risk the divergence of MCMC chains towards inaccurate posterior estimates, if inaccurate initial values are used. We note that inaccurate priors can yield inaccurate and imprecise inference. Bounded priors should certainly not be used unless their accuracy is well established. Inaccurate estimates of sensitivity or specificity can yield wildly inaccurate estimates of disease prevalence. Our analyses are motivated by studies of bovine tuberculosis in a wild badger population.

## Introduction

Uncertainty lies at the heart of real-world epidemiology. While hosts might be truly infected or uninfected, and diseased or not, our observation of these states suffers from imperfect detection of hosts, infection and disease. Ecological studies tend to deal with imperfect host detection using capture-mark-recapture methodologies [e.g., (1)], with limited consideration of biases in diagnoses themselves [but see (2)]. However, imperfect pathogen detection is a common occurrence when sampling live populations, with studies often drawing conclusions from the results of one or more tests, none of which are 100% accurate [e.g., (3)]. This is important because methods for the accurate detection of disease are pivotal to surveillance programmes that focus on the spatial and temporal spread of pathogens within and between populations, with infection prevalence often the primary parameter of interest (4–7).

For many wildlife diseases, post-mortem analysis provides gold standard diagnosis, however this is not an option for most ecological studies of wild animals, where corpses are hard to find and where an understanding of natural disease dynamics is the primary goal. Given the rarity of gold standard diagnostics for live animals, the development of statistical approaches for the evaluation of imperfect diagnostic tests has been an active field of research applied to human [e.g., (8)] and veterinary medicine [e.g., (9)]. These approaches can account for misclassification of both test positive individuals and test negative individuals. Specifically, they quantify test sensitivity, which measures the probability of a positive test outcome caused by the individual having the disease (the probability of a true positive outcome), and test specificity, which measures the probability of a negative test caused by the individual not having the disease (the probability of a true negative outcome).

Accounting for test sensitivity and specificity is vital to the accurate estimation of disease prevalence in a host population. Bayesian latent class analysis evaluates the performance of diagnostic tests in the absence of a reference test (10, 11) and consequently provides suitably adjusted estimates of prevalence. The development of Bayesian approaches for diagnostic test evaluation now provides a means to simultaneously estimate the performance of multiple tests in the light of the others and provide accurate estimates of disease prevalence that accounts for diagnostic uncertainty (12). A desirable aspect of Bayesian analysis is the pairing of data with prior information to estimate parameters that may have been previously unidentifiable, offering practical advantages over frequentist approaches (13). Despite these obvious benefits, often little is known about test performance in the field and the determination of an informative prior can be a challenging and subjective process. Although we know the choice of a prior contributes to the posterior distribution (12), it remains unclear how such prior sensitivity affects our inference regarding disease prevalence.

Here we explore how the accuracy and precision of prior information influence conclusions regarding disease prevalence. Our objectives are threefold. First, using simulated data for three diagnostic tests based on a previous analysis of bovine tuberculosis in a wild population of European badgers (9), we ask: (1) how does parameter identifiability compare between models that use precise (informative) and imprecise (vague or uninformative) priors? (2) How do inaccurate priors for test sensitivity and specificity influence conclusions regarding overall prevalence? (3) How accurate are prevalence estimates that rely on the assumption that specificity and sensitivity are both perfect (i.e., both = 1)? From an applied perspective, disentangling disease processes from test performance will aid in forecasting long-term dynamics and in developing control strategies. This is especially important as diagnostic uncertainty has the potential to hinder eradication efforts by masking or exaggerating observed disease patterns. However, without robust statistical approaches it is impossible to understand how such bias may influence prevalence estimates.

## Materials and Methods

### Simulated Data

We simulated results of three independent diagnostic tests for 875 individuals, using the prior modes of prevalence, specificity and sensitivity taken from Drewe et al. (9) (Table 1). The observations lead to a cross-classification table for the joint test results **y** = (y_111_, y_121_, y_112_, y_122_, y_211_, y_221_, y_212_, y_222_) where y_111_ is number of sampled individuals that tested positive for all three diagnostic tests and y_222_ is number of sampled individuals that tested negative for all three diagnostic tests.

**Table 1:** Simulated values taken from Drewe et al. (9) along with prior distributions used for precise, vague and badly stated priors.

**Test**	**Parameter**	**Simulated values**	**Prior distributions on the logit scale ** **Normal (µ, σ)**					
			**Precise**	**Vague**	**Error**	**Error**	**Error**	**Error**
**Test A**	**Sensitivity**	0.492	−0.032, 0.125	0, 1.65	−**0.847, 0.162**	**0.809, 0.139**	−0.032, 0.125	−0.032, 0.125
	**Specificity**	0.931	2.602, 1.073	0, 1.65	2.602, 1.073	2.602, 1.073	2.602, 1.073	2.602, 1.073
**Test B**	**Sensitivity**	0.809	1.443, 0.443	0, 1.65	1.443, 0.443	1.443, 0.443	1.443, 0.443	1.443, 0.443
	**Specificity**	0.936	2.683, 1.117	0, 1.65	2.683, 1.117	2.683, 1.117	**1.025, 0.271**	**6.907, ** **1.179**
**Test C**	**Sensitivity**	0.100	−2.197, 0.856	0, 1.65	−2.197, 0.856	−2.197, 0.856	−2.197, 0.856	−2.197, 0.856
	**Specificity**	0.999	4.595, 2.129	0, 1.65	4.595, 2.129	4.595, 2.129	4.595, 2.129	4.595, 2.129
	**Prevalence**	0.24	−1.153, 0.272	0, 1.65	−1.153, 0.272	−1.153, 0.272	−1.153, 0.272	−1.153, 0.272

**Figure 1:**
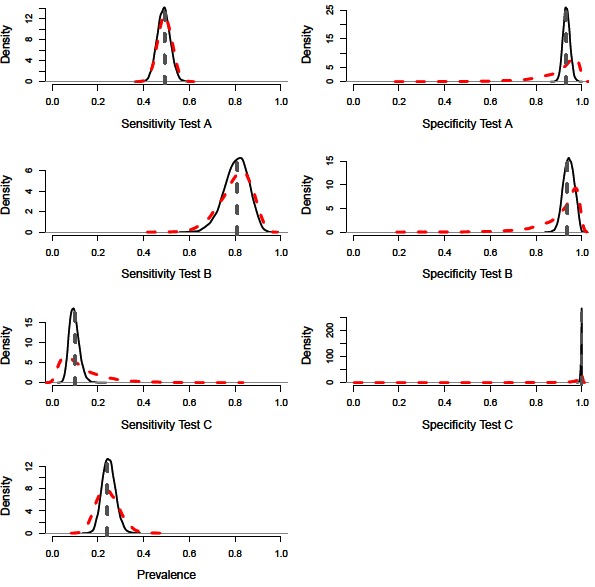
Distributions for sensitivity and specificity of diagnostic tests, alongside prevalence estimates from a constant model. Showing the precise prior distributions (red-dashed line), the posterior distribution (black-solid lines) and simulated mean value (grey-dashed line).

**Figure 2:**
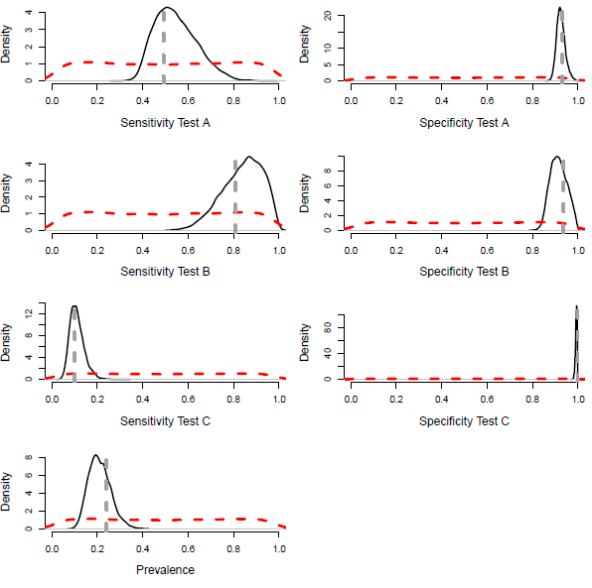
Distributions for sensitivity and specificity of diagnostic tests, alongside prevalence estimates from a constant model. Showing the vague prior distributions (red-dashed line), the posterior distribution (black-solid lines) and simulated mean value (grey-dashed line).

Badly stated priors are precise but inaccurate for one of the sensitivities or specificities of the tests. These inaccuracies are emboldened in the body of the table. “Precise” priors are chosen to be precise on the probability scale, which means that canonical links between mean and variance can make them look imprecise on the logit scale when the mean is near zero or one. We provide the back-transformation of these priors onto the probability scale as red dashed lines in Figures 1 and 2.

### Assessment of Prevalence Estimation

The parameters for the model include the three sensitivities (Se), three specificities (Sp) and prevalence (π). Biological independence between tests was assumed. Multinomial cell probabilities for the population are given by:

$$\text{\textbackslash{}[}\begin{array}{l} y∼multinomial(n,p_{jjj}),j\in \{1,2\}\\ p_{111}=\pi Se_{A}Se_{B}Se_{C}+(1-\pi )(1-Sp_{A})(1-Sp_{B})(1-Sp_{C})\\ p_{121}=\pi Se_{A}(1-Se_{B})Se_{C}+(1-\pi )(1-Sp_{A})Sp_{B}(1-Sp_{C})\\ p_{112}=\pi Se_{A}Se_{B}(1-Se_{C})+(1-\pi )(1-Sp_{A})(1-Sp_{B})Sp_{C}\\ p_{122}=\pi Se_{A}(1-Se_{B})(1-Se_{C})+(1-\pi )(1-Sp_{A})Sp_{B}Sp_{C}\\ p_{211}=\pi Se_{A}Se_{B}(1-Se_{C})+(1-\pi )Sp_{A}Sp_{B}(1-Sp_{C})\\ p_{221}=\pi (1-Se_{A})(1-Se_{B})Se_{C}+(1-\pi )Sp_{A}Sp_{B}(1-Sp_{C})\\ p_{212}=\pi (1-Se_{A})Se_{B}(1-Se_{C})+(1-\pi )Sp_{A}(1-Sp_{B})Sp_{C}\\ p_{222}=\pi (1-Se_{A})(1-Se_{B})(1-Se_{C})+(1-\pi )Sp_{A}Sp_{B}Sp_{C} \end{array}\text{\textbackslash{}]}$$

where *j* = 1 describes a positive test outcome, and *j* = 2 describes a negative test outcome for each of the three tests A, B and C. Prevalence *π* was set at 0.24.

To ensure parameters were bounded between 0 and 1 we modelled the logit of sensitivities, specificities and prevalence using Normal prior distributions with mean µ and SD σ. We compared precise priors (small σ, implying strong prior knowledge) with vague priors (large σ, implying weak prior knowledge). Inaccurate but precise priors (implying strong prior belief in a wrong parameter value) were also incorporated to explore the influence of misinformed beliefs on inference of disease prevalence. Priors for the different modelling scenarios are shown in Table 1.

### Study System

Our simulations of test specificity and disease prevalence are based on the long term study of natural epidemiology of *M. bovis* among wild European badgers in Woodchester Park, Gloucestershire, UK. Here, badgers are regularly live trapped, sampled using diagnostic tests, and released (14). Three diagnostic tests were used routinely during the period assessed by Drewe et al. (9) to assess the infection status of each trapped individual. Blood samples are taken to test for antibodies to *M. bovis* using Stat-Pak (simulated as Test A), and further used to test for a cell-mediated response to *M. bovis* using interferon-gamma (IFNg; Test B). Samples of faeces, urine, tracheal aspirate, oesophageal aspirate and swabs from bite wounds (where present) are collected for mycobacterial culture (Test C). Estimates of specificity and sensitivity of each of the diagnostic tests, used here to inform our simulations, are drawn from Drewe et al. (9), although these were subsequently updated by Buzdugan et al. (15). Given this description of the study system, it is difficult to justify the assumption of independence of test outcomes made by our simulations; tests A & B are applied to the same blood samples; the test sensitivities and specificities depend on different stages of disease progression in infected individuals. This, combined with the importance of the badger-bTB system, is why we emphasise that this analysis is motivated by, but not definitive for, theprevalence of bovine tuberculosis in badgers. A definitive analysis would have to account for non-independence of test outcomes, and for longitudinal patterns of disease progression within host individuals.

### Model Fitting

We fit all models using Bayesian methods and estimated the posterior distributions for all parameters using MCMC implemented in winBUGS (16) with the R2Winbugs package (17) in R (18). Convergence was assessed both visually ensuring mixing of the chains and formally within the model calculating the potential scale reduction factor ($\hat{R}$). When $\hat{R}$ is close to one we can be confident that convergence has been reached (19). Consequently, posterior distributions were computed after a burn in of 5,000, followed by 50,000 iterations with a thinning interval of 10 iterations. WinBUGS code is provided as supplementary material.

## Results

### Simulation Summary

By simulating data, we determine whether prevalence is estimated accurately (i.e., the true value should lie within 95% bounds of credibility of the posterior estimate) and precisely (i.e., the 95% bounds of credibility are usefully tight around the posterior estimate), using (1) raw test outcomes assuming perfect sensitivity and specificity, (2) models with incorrect, precise priors, (3) models with accurate, precise priors and (4) models with imprecise (vague) priors.

### Simulated Population

**Figure 3:**
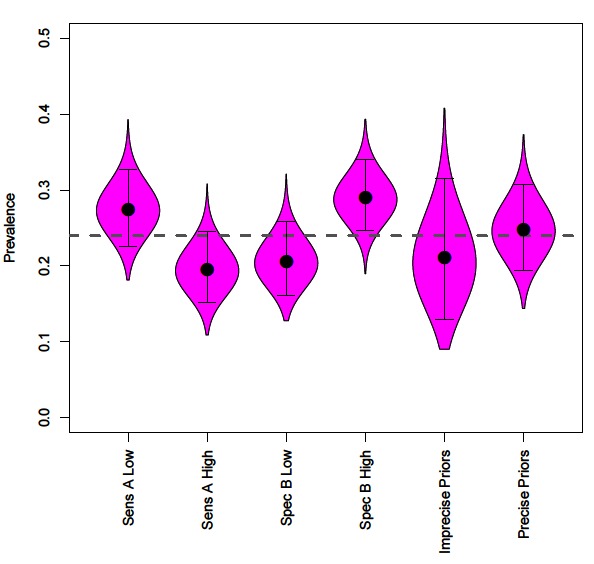
The effect of incorrectly specifying priors on disease prevalence estimates, compared to mean prevalence (grey dashed line). Along with prevalence obtained by uninformative and informative priors for all parameters.

A constant Bayesian model (i.e., assuming no variation in epidemiological or diagnostic parameters through time; (9, 11) with precise priors successfully estimated the diagnostic performance values and disease prevalence of the simulated population (Figure 1). The model also performed accurately with imprecise priors, although the posterior distributions were less precise, accounting for the additional uncertainty (Figures 2 and 3, Table 2). However, we note that with imprecise priors, the chains required realistic initial values to ensure convergence. Parallel chains occurred in models that excluded informative priors and included randomly assigned initial values, indicating more than one area of high posterior probability. However, with realistic initial starting values for the chains all parameters were identifiable, despite vague priors (Figures 2 and 3).

**Table 2:** Prevalence estimates derived from raw outcomes of each test (assuming perfect sensitivity and specificity), and inferred using latent class analysis, along with 95% credible intervals.

**Method to estimate prevalence**	**Prevalence**
*Assume perfect diagnostics*	
Test A	0.170
Test B	0.242
Test C	0.025
*Modelling diagnostic uncertainty*	Mean (95% Credible Interval)
Precise priors	0.248 (0.194, 0.308)
Vague priors	0.209 (0.129, 0.311)
*Incorrect prior specification*	
Reduced sensitivity test A	0.274 (0.226, 0.327)
Increased sensitivity test A	0.196 (0.152, 0.249)
Reduced specificity test B	0.206 (0.161, 0.259)
Increased specificity test B	0.289 (0.247, 0.338)

The true value of prevalence in our simulation is 0.24.

Precise priors can aid identifiability, however with often limited information regarding the performance of diagnostic tests we explore how the accuracy of the prior information impacts conclusions regarding disease prevalence using four scenarios; (1) prior specifies lower test A sensitivity (µ = 0.292); (2) prior specifies higher test A sensitivity (µ = 0.692); (3) prior specifies lower test B specificity (µ = 0.736); (4) prior specifies higher test B specificity (µ = 0.999).

Incorrect specification of priors leads to inaccurate prevalence estimates (Figure 3). Prior information that reduces the sensitivity of a test leads to assumptions of more false negatives and consequently increased prevalence estimates (Figure 3). In contrast, prior information that increases the sensitivity of a test leads to assumptions of fewer false negatives and decreased prevalence estimates (Figure 3).

Incorrect priors on specificity values changes assumptions regarding false positives. Reduced test specificity, caused by badly specified priors, infers higher numbers of false-positives and therefore reduces estimates of disease prevalence (Figure 3). The flipside of this is that increased test specificity assumes lower rates of false positives and increases estimates of prevalence (Figure 3).

Raw values from diagnostic tests with low sensitivity produced inaccurate estimates of prevalence when perfect diagnoses were assumed (Table 2). We find that test B, which has high relative sensitivity and specificity, and therefore the lowest number of false negatives and low numbers of false positives, naturally provided the most accurate estimate of prevalence (Table 2).

## Discussion

### Methodological Guidance

*Raw diagnostic outcomes should not be used to infer prevalence.* In the absence of knowledge regarding sensitivity and specificity of the underlying tests, using the raw test results can provide highly inaccurate estimates of prevalence.

*To be certain of convergence run multiple chains*. From the simulations we have ascertained that parameters are identifiable despite vague priors. However, realistic starting values are required as chains can get stuck in different regions, indicating a bottleneck between two regions of high probability, or multiple posterior modes. The occurrence of multiple posterior modes would not be identified without running multiple chains. If realistic initial values are unknown, then inference of the “true” state should be made with caution.

*Beware precise priors*. Inaccurate, precise priors can bias parameter estimates and can lead to inaccurate conclusions regarding prevalence. This will be particularly true if priors are bounded above and/or below, for example if using Uniform prior distributions. Diagnosis of such inaccuracy might be possible if posterior distributions are clustered at either bound, however we recommend the avoidance of bounded priors unless prior knowledge is strong and accurate.

### General Discussion

Disease prevalence is fundamental to our understanding of wildlife epidemiology and is often the focal parameter when it comes to deriving management recommendations. However, the accuracy of diagnostics used to live sample wild populations is often uncertain and has the potential to alter conclusions regarding the prevalence of disease. Indeed, we have shown the importance of accounting for bias in determining disease parameters within wild populations, with raw data usually providing a poor representation of the true prevalence. Using a simulated population, we demonstrated how Bayesian latent class analysis can provide accurate estimates of test performance and infection prevalence in the absence of a reference test. However, this method is not without its caveats as poorly specified priors can heavily influence the posterior, altering conclusions regarding prevalence of infection.

Prevalence only provides a reliable surveillance indicator if issues of diagnostic uncertainty are accounted for. As our study shows, tests that have low sensitivities can vastly underestimate prevalence, due to the inclusion of high numbers of false negative individuals. Further, inaccuracies in prior information on specificity can yield inaccurate inference of credible ranges of prevalence, due to poor inference of false positive diagnoses. In the absence of knowledge regarding specificity and sensitivity, some ecological studies attempt to minimise the risk of misclassification by focusing on individuals that are diseased rather than infected, because disease tends to be accompanied by visible symptoms (2, 20). While this approach minimises false positives, it accepts that false negatives can occur and risks the underestimation of true prevalence. We also note that estimates of prevalence based on raw outcomes of diagnostic tests can also be useful for longitudinal studies of “relative” prevalence, or for comparisons among populations [e.g., (6)].

Latent class analysis provides a solution, and has been used extensively to estimate both sensitivities and specificities of diagnostic tests in the absence of a reference test across a range of diseases (9–11, 21). A prerequisite of Bayesian analysis is the combination of information from data and prior information. Commonly, when there is a lack of information surrounding parameters, vague priors are used to ensure the posterior distributions are driven by the data alone. This is advisable given we find posteriors to be sensitive to the selection of priors, similar to findings from previous studies (12). However, when a model is not identifiable, for example when there are more parameters than degrees of freedom, constraints or/and informative prior distributions are required to obtain a computational solution (8). When required for identifiability issues, informative priors should be used cautiously and only on parameters with a strong knowledge base. In these scenarios multiple chains are recommended to check for prior sensitivity. Additionally, in our simulated example (7 parameters, 7 degrees of freedom), we find parameters are identifiable but suffer from convergence issues if initial values are randomly assigned. This indicates more than one area of high posterior probability. Using precise initial values enables convergence in one area of parameter space, but accuracy can only be assumed if the initial values are themselves accurate. However, when there is a complete lack of knowledge then application of different starting values to explore differing areas of high posterior probability will be required.

Heterogeneity in test performance across tests and populations are commonly incorporated within the Bayesian approach presented here (11). The modelling we have performed did not consider variation in test performance and prevalence of infection through time. We recommend further development of latent class analysis to incorporate variation or trends in sensitivity, specificity and prevalence through time. Similar to the benefits of extending data input across populations (11), an advantage of incorporating time-varying data on diagnostic outcomes is an increase in the degrees of freedom provided by decomposition of diagnostic outcomes into timesteps. The cost of such a decomposition will be a reduction in the sample size of individuals diagnosed per timestep. As well as variation through space and time, diagnostic outcomes will vary according to the circumstances of the individual being tested. Detecting the clinical status of the host may depend on a range of additional factors such as their age, sex, coinfections, disease severity or stress. For example, pathogen detection may vary as a function of pathogen load, with seropositivity associated with advanced stages of disease (22). Reconciling links between both individual and population level test performance in an integrated framework is an important area of future research.

The choice of latent class model, for the estimation of diagnostic test performance and infection prevalence, will always depend on the purpose of the study. Latent class analysis, or “diagnosis without gold standard” relies on the use of multiple diagnostic tests to infer sensitivity, specificity and prevalence. If the performance of diagnostic tests is the reason for study, then it makes sense to study multiple populations that vary in prevalence, or a single population that varies through time, with one reliable test to use as a standard. If tests cannot be assumed to be independent, then covariance must be modelled and more tests or replicate populations are required to cope with the demand on degrees of freedom. Mostly, in studies of wild host populations, there is no gold standard and little knowledge of test dependencies, and the simple approach we expound here is a good starting point. We have only simulated a single population to make our point about precision and accuracy, but the supplementary code (Data Sheet S1) we provide allows interested readers to explore various scenarios. A benefit of BUGS code is that it can be used as a building block for more complex modelling scenarios.

## Conclusion

Understanding links between diagnostic uncertainty and prevalence provides a key to explaining and predicting the population dynamics of infected hosts, and ultimately informs the development, and tests the efficacy, of management for disease control. We demonstrate the utility of Bayesian latent class analysis, developed to assess diagnostic sensitivity and specificity in the absence of gold standard tests. Analysis revealed complexities underpinning misclassification bias, including inaccuracy and imprecision of priors, which fundamentally influence our understanding of disease dynamics within wildlife-host systems. Increasingly epidemiological data is available at population and temporal scales necessary to estimate diagnostic parameters. We therefore recommend further model development and ultimately application of this approach to surveys in other populations and species.

## Author Contributions

JM coded the models and drafted the paper. DH motivated the research, re-analysed the models and write the final draft.

## Conflict of interest statement

The authors declare that the research was conducted in the absence of any commercial or financial relationships that could be construed as a potential conflict of interest.
