# Supplementary material for: Prior Precision, Prior Accuracy, and the Estimation of Disease Prevalence Using Imperfect Diagnostic Tests
Source: Front Vet Sci. 2018 May 11;5:83. doi: 10.3389/fvets.2018.00083 (PMC5958675; doi:10.3389/fvets.2018.00083)
Supplement: Supplementary file 1 [file DataSheet1.DOCX]

library(R2WinBUGS)

bugs.dir<- "C:/WinBUGS14"

###SIMULATE CONSTANT PREV AND DIAGNOSTIC TEST SENS/SPEC

#####create prevalence using posterior modes from Drewe et al 2010

#Culture Se 0.100 Sp 0.999

#IFN Se 0.809 Sp 0.936

#Stat-Pak Se 0.492 Sp 0.931

SeC<-0.100

SpC<-0.999

SeI<-0.809

SpI<-0.936

SeS<-0.492

SpS<-0.931

prev<-0.24

#my.array[1,,]

my.array <- array(0, dim=c(2,2,2),dimnames=list(Gamma=c("positive","Negative"),Statpak=c("Postive","Negative"),Culture=c("Positive","Negative")))

n=875

sum(my.array)

my.array <- array(0, dim=c(2,2,2),dimnames=list(Gamma=c("positive","Negative"),Statpak=c("Postive","Negative"),Culture=c("Positive","Negative")))

n=875

#Culture Pos

my.array[1,1,1]<-round(n*(( (prev*SeC*SeI*SeS) + ((1-prev)*(1-SpC)*(1-SpI)*(1-SpS)) )))

my.array[2,1,1]<-round(n*(( prev*SeC*(1-SeI)*SeS) + ((1-prev)*(1-SpC)*(SpI)*(1-SpS) )))

my.array[1,2,1]<-round(n*((( prev*SeC*SeI*(1-SeS)) + ((1-prev)*(1-SpC)*(1-SpI)*(SpS)) )))

my.array[2,2,1]<-round(n*((( prev*SeC*(1-SeI)*(1-SeS)) + ((1-prev)*(1-SpC)*(SpI)*(SpS)) ) ))

#Culture Neg

my.array[1,1,2]<-round(n*(( (prev*(1-SeC)*SeI*SeS) + ((1-prev)*(SpC)*(1-SpI)*(1-SpS)) )))

my.array[2,1,2]<-round(n*(( prev*(1-SeC)*(1-SeI)*SeS) + ((1-prev)*(SpC)*(SpI)*(1-SpS) )))

my.array[1,2,2]<-round(n*((( prev*(1-SeC)*SeI*(1-SeS)) + ((1-prev)*(SpC)*(1-SpI)*(SpS)) )))

my.array[2,2,2]<-round(n*((( prev*(1-SeC)*(1-SeI)*(1-SeS)) + ((1-prev)*(SpC)*(SpI)*(SpS)) ) ))

sum(my.array)

##Prevalence based on raw values

##based on IFN test

sum(my.array[1,,])/n #0.2422857 prevalence

##based on Stat-Pak test

sum(my.array[,1,])/n #] 0.1702857 prevalence

##based on Culture test

sum(my.array[,,1])/n #] 0.02514286 prev

#######INFORMATIVE & ACCURATE PRIORS

sink("winbugsmodel2.bug")

cat("

##model

## Cu=culture, In=IFNg, St=StatPak, Se=sensitivity, Sp=specificity, Pi=prevalence

model {

y[1:K, 1:K, 1:K] ~ dmulti(p[1:K, 1:K, 1:K], n)

p[1,1,1] <- pi*SeCu*(SeIn*SeSt) + (1-pi)*(1-SpCu)*((1-SpIn)*(1-SpSt))

p[1,2,1] <- pi*SeCu*(SeIn*(1-SeSt)) + (1-pi)*(1-SpCu)*((1-SpIn)*SpSt)

p[1,1,2] <- pi*(1-SeCu)*(SeIn*SeSt) + (1-pi)*SpCu*((1-SpIn)*(1-SpSt))

p[1,2,2] <- pi*(1-SeCu)*(SeIn*(1-SeSt)) + (1-pi)*SpCu*((1-SpIn)*SpSt)

p[2,1,1] <- pi*SeCu*((1-SeIn)*SeSt) + (1-pi)*(1-SpCu)*(SpIn*(1-SpSt))

p[2,2,1] <- pi*SeCu*((1-SeIn)*(1-SeSt)) + (1-pi)*(1-SpCu)*(SpIn*SpSt)

p[2,1,2] <- pi*(1-SeCu)*((1-SeIn)*SeSt) + (1-pi)*SpCu*(SpIn*(1-SpSt))

p[2,2,2] <- pi*(1-SeCu)*((1-SeIn)*(1-SeSt)) + (1-pi)*SpCu*(SpIn*SpSt)

SeIn<-1/(1+exp(-S.SeIn))

S.SeIn ~ dnorm(1.443525,5.09696)

SpIn<-1/(1+exp(-S.SpIn))

S.SpIn ~ dnorm( 2.682732,0.8017619)

SeSt<-1/(1+exp(-S.SeSt))

S.SeSt ~ dnorm(-0.03200273,63.5998)

SpSt<-1/(1+exp(-S.SpSt))

S.SpSt ~ dnorm( 2.602153, 0.8677139)

SeCu<-1/(1+exp(-S.SeCu))

S.SeCu ~ dnorm(-2.197225,1.364591)

SpCu<-1/(1+exp(-S.SpCu))

S.SpCu ~ dnorm(4.59512, 0.2206248 )

pi<-1/(1+exp(-S.pi))

S.pi ~ dnorm(-1.15268,13.49009)

}

",fill = TRUE)

sink()

# Bundle data

bugs.data <- list(y= my.array,K=2,n=875)

# Initial values

inits <- function(){list(pi=0.24, SeIn=0.81, SpIn=0.94, SeSt=0.49, SpSt=0.93, SeCu=0.10, SpCu=1.00)}

# Parameters monitored

parameters <- c("pi","SeIn","SpIn","SeSt","SpSt","SeCu","SpCu")

# MCMC settings

ni <- 50000

nt <- 10

nb <- 5000

nc <- 3

# Call WinBUGS from R (BRT 1 min)

Inform <- bugs(bugs.data, inits=NULL, parameters, "winbugsmodel2.bug", n.chains = nc, n.thin = nt, n.iter = ni, n.burnin = nb, debug = FALSE, bugs.directory = bugs.dir, working.directory = getwd())

print(Inform,digits=3)

#Plot comparing priors with posteriors

pdf("Fig1 priors vs posteriors.pdf")

par(mfrow=c(4,2),mar=c(5,4,1,1)+0.1,cex.lab=1.2)

plot(density(((Inform$sims.list$SeSt))),xlim=c(0.01,0.99),lwd=2,main="",bty='n',xlab="Sensitivity Test A")

gaus<-rnorm(10000,-0.03200273,sqrt(1/63.5998))

lines(density(inv.logit(gaus)),xlim=c(0.4,0.75),ylim=c(0,20),col="red",lwd=3,lty=2)

abline(v=0.492,lty=2,lwd=3,col="grey60")

plot(density(((Inform$sims.list$SpSt))),xlim=c(0.01,0.99),lwd=2,main="",bty='n',xlab="Specificity Test A")

gaus<-rnorm(10000,2.602153,sqrt(1/0.8677139))

lines(density(inv.logit(gaus)),xlim=c(0.4,0.75),ylim=c(0,20),col="red",lwd=3,lty=2)

abline(v=0.931,lty=2,lwd=3,col="grey60")

plot(density(((Inform$sims.list$SeIn))),xlim=c(0.01,0.99),lwd=2,main="",bty='n',xlab="Sensitivity Test B")

gaus<-rnorm(10000,1.443525,sqrt(1/5.09696))

lines(density(inv.logit(gaus)),xlim=c(0.4,0.75),ylim=c(0,20),col="red",lwd=3,lty=2)

abline(v=0.809,lty=2,lwd=3,col="grey60")

plot(density(((Inform$sims.list$SpIn))),xlim=c(0.01,0.99),lwd=2,main="",bty='n',xlab="Specificity Test B")

gaus<-rnorm(10000,2.682732,sqrt(1/0.8017619))

lines(density(inv.logit(gaus)),xlim=c(0.4,0.75),ylim=c(0,20),col="red",lwd=3,lty=2)

abline(v=0.936,lty=2,lwd=3,col="grey60")

plot(density(((Inform$sims.list$SeCu))),xlim=c(0.01,0.99),lwd=2,main="",bty='n',xlab="Sensitivity Test C")

gaus<-rnorm(10000,-2.197225,sqrt(1/1.364591))

lines(density(inv.logit(gaus)),xlim=c(0.4,0.75),ylim=c(0,20),col="red",lwd=3,lty=2)

abline(v=0.1,lty=2,lwd=3,col="grey60")

plot(density(((Inform$sims.list$SpCu))),xlim=c(0.01,0.99),lwd=2,main="",bty='n',xlab="Specificity Test C")

gaus<-rnorm(10000,4.59512,sqrt(1/0.2206248))

lines(density(inv.logit(gaus)),xlim=c(0.4,0.75),ylim=c(0,20),col="red",lwd=3,lty=2)

abline(v=0.999,lty=2,lwd=3,col="grey60")

plot(density(((Inform$sims.list$pi))),xlim=c(0.01,0.99),lwd=2,main="",bty='n',xlab="Prevalence")

gaus<-rnorm(10000,-1.15268,sqrt(1/13.49009))

lines(density(inv.logit(gaus)),xlim=c(0.4,0.75),ylim=c(0,20),col="red",lwd=3,lty=2)

abline(v=0.24,lty=2,lwd=3,col="grey60")

dev.off()

############################################################################

###INCORRECTLY SPECIFYING PRIORS

### First reduce sensitivity of Test A

###### lower it first to 0.292(0.231,0.453)#Stat-Pak Se 0.492 (0.431, 0.553)Drewe et al 2010

mu=logit(0.3)

sigma=(logit(0.292)-logit(0.231))/1.96#as this is 95% CI

1/( sigma^2)#tau= 38.23317

mu# -0.8472979

gaus<-rnorm(1000,mu,sigma)

plot(density(inv.logit(gaus)))

#######INFORM

sink("winbugsmodel2.bug")

cat("

##model

## Cu=culture, In=IFNg, St=StatPak, Se=sensitivity, Sp=specificity, Pi=prevalence

model {

y[1:K, 1:K, 1:K] ~ dmulti(p[1:K, 1:K, 1:K], n)

p[1,1,1] <- pi*SeCu*(SeIn*SeSt) + (1-pi)*(1-SpCu)*((1-SpIn)*(1-SpSt))

p[1,2,1] <- pi*SeCu*(SeIn*(1-SeSt)) + (1-pi)*(1-SpCu)*((1-SpIn)*SpSt)

p[1,1,2] <- pi*(1-SeCu)*(SeIn*SeSt) + (1-pi)*SpCu*((1-SpIn)*(1-SpSt))

p[1,2,2] <- pi*(1-SeCu)*(SeIn*(1-SeSt)) + (1-pi)*SpCu*((1-SpIn)*SpSt)

p[2,1,1] <- pi*SeCu*((1-SeIn)*SeSt) + (1-pi)*(1-SpCu)*(SpIn*(1-SpSt))

p[2,2,1] <- pi*SeCu*((1-SeIn)*(1-SeSt)) + (1-pi)*(1-SpCu)*(SpIn*SpSt)

p[2,1,2] <- pi*(1-SeCu)*((1-SeIn)*SeSt) + (1-pi)*SpCu*(SpIn*(1-SpSt))

p[2,2,2] <- pi*(1-SeCu)*((1-SeIn)*(1-SeSt)) + (1-pi)*SpCu*(SpIn*SpSt)

SeIn<-1/(1+exp(-S.SeIn))

S.SeIn ~ dnorm(1.443525,5.09696)

SpIn<-1/(1+exp(-S.SpIn))

S.SpIn ~ dnorm( 2.682732,0.8017619)

SeSt<-1/(1+exp(-S.SeSt))

S.SeSt ~ dnorm(-0.8472979,38.23317)##Incorrect prior

SpSt<-1/(1+exp(-S.SpSt))

S.SpSt ~ dnorm( 2.602153, 0.8677139)

SeCu<-1/(1+exp(-S.SeCu))

S.SeCu ~ dnorm(-2.197225,1.364591)

SpCu<-1/(1+exp(-S.SpCu))

S.SpCu ~ dnorm(4.59512, 0.2206248 )

pi<-1/(1+exp(-S.pi))

S.pi ~ dnorm(-1.15268,13.49009)

}

",fill = TRUE)

sink()

# Bundle data

bugs.data <- list(y= my.array,K=2,n=875)

# Initial values

inits <- function(){list(pi=0.24, SeIn=0.81, SpIn=0.94, SeSt=0.49, SpSt=0.93, SeCu=0.10, SpCu=1.00)}

# Parameters monitored

parameters <- c("pi","SeIn","SpIn","SeSt","SpSt","SeCu","SpCu")

# MCMC settings

ni <- 50000

nt <- 10

nb <- 5000

nc <- 3

# Call WinBUGS from R (BRT 1 min)

Inform_incorrect_1 <- bugs(bugs.data, inits=NULL, parameters, "winbugsmodel2.bug", n.chains = nc, n.thin = nt, n.iter = ni, n.burnin = nb, debug = FALSE, bugs.directory = bugs.dir, working.directory = getwd())

print(Inform_incorrect_1,digits=3)

#Plot comparing priors with posteriors

pdf("Fig TestA sens low.pdf")

par(mfrow=c(4,2),mar=c(5,4,1,1)+0.1,cex.lab=1.2)

plot(density(((Inform_incorrect_1$sims.list$SeSt))),xlim=c(0.01,0.99),lwd=3,main="",bty='n',xlab="Sensitivity Test A")

gaus<-rnorm(10000,-0.8472979,sqrt(1/38.23317))

lines(density(inv.logit(gaus)),xlim=c(0.4,0.75),ylim=c(0,20),col="red",lwd=3,lty=2)

abline(v=0.492,lty=2,lwd=3,col="grey60")

plot(density(((Inform_incorrect_1$sims.list$SpSt))),xlim=c(0.01,0.99),lwd=3,main="",bty='n',xlab="Specificity Test A")

gaus<-rnorm(10000,2.602153,sqrt(1/0.8677139))

lines(density(inv.logit(gaus)),xlim=c(0.4,0.75),ylim=c(0,20),col="red",lwd=3,lty=2)

abline(v=0.931,lty=2,lwd=3,col="grey60")

plot(density(((Inform_incorrect_1$sims.list$SeIn))),xlim=c(0.01,0.99),lwd=3,main="",bty='n',xlab="Sensitivity Test B")

gaus<-rnorm(10000,1.443525,sqrt(1/5.09696))

lines(density(inv.logit(gaus)),xlim=c(0.4,0.75),ylim=c(0,20),col="red",lwd=3,lty=2)

abline(v=0.809,lty=2,lwd=3,col="grey60")

plot(density(((Inform_incorrect_1$sims.list$SpIn))),xlim=c(0.01,0.99),lwd=3,main="",bty='n',xlab="Specificity Test B")

gaus<-rnorm(10000,2.682732,sqrt(1/0.8017619))

lines(density(inv.logit(gaus)),xlim=c(0.4,0.75),ylim=c(0,20),col="red",lwd=3,lty=2)

abline(v=0.936,lty=2,lwd=3,col="grey60")

plot(density(((Inform_incorrect_1$sims.list$SeCu))),xlim=c(0.01,0.99),lwd=3,main="",bty='n',xlab="Sensitivity Test C")

gaus<-rnorm(10000,-2.197225,sqrt(1/1.364591))

lines(density(inv.logit(gaus)),xlim=c(0.4,0.75),ylim=c(0,20),col="red",lwd=3,lty=2)

abline(v=0.1,lty=2,lwd=3,col="grey60")

plot(density(((Inform_incorrect_1$sims.list$SpCu))),xlim=c(0.01,0.99),lwd=3,main="",bty='n',xlab="Specificity Test C")

gaus<-rnorm(10000,4.59512,sqrt(1/0.2206248))

lines(density(inv.logit(gaus)),xlim=c(0.4,0.75),ylim=c(0,20),col="red",lwd=3,lty=2)

abline(v=0.999,lty=2,lwd=3,col="grey60")

plot(density(((Inform_incorrect_1$sims.list$pi))),xlim=c(0.01,0.99),lwd=3,main="",bty='n',xlab="Prevalence")

gaus<-rnorm(10000,-1.15268,sqrt(1/13.49009))

lines(density(inv.logit(gaus)),xlim=c(0.4,0.75),ylim=c(0,20),col="red",lwd=3,lty=2)

abline(v=0.24,lty=2,lwd=3,col="grey60")

dev.off()

##################################################################################

### Second increase sensitivity of Test A

###### lower it first to 0.692(0.631,0.753)#Stat-Pak Se 0.492 (0.431, 0.553)Drewe et al 2010

mu=logit(0.692)

sigma=(logit(0.692)-logit(0.631))/1.96#as this is 95% CI

1/( sigma^2)#tau= 51.55374

mu# 0.8094862

gaus<-rnorm(1000,mu,sigma)

plot(density(inv.logit(gaus)))

#######INFORM

sink("winbugsmodel2.bug")

cat("

##model

## Cu=culture, In=IFNg, St=StatPak, Se=sensitivity, Sp=specificity, Pi=prevalence

model {

y[1:K, 1:K, 1:K] ~ dmulti(p[1:K, 1:K, 1:K], n)

p[1,1,1] <- pi*SeCu*(SeIn*SeSt) + (1-pi)*(1-SpCu)*((1-SpIn)*(1-SpSt))

p[1,2,1] <- pi*SeCu*(SeIn*(1-SeSt)) + (1-pi)*(1-SpCu)*((1-SpIn)*SpSt)

p[1,1,2] <- pi*(1-SeCu)*(SeIn*SeSt) + (1-pi)*SpCu*((1-SpIn)*(1-SpSt))

p[1,2,2] <- pi*(1-SeCu)*(SeIn*(1-SeSt)) + (1-pi)*SpCu*((1-SpIn)*SpSt)

p[2,1,1] <- pi*SeCu*((1-SeIn)*SeSt) + (1-pi)*(1-SpCu)*(SpIn*(1-SpSt))

p[2,2,1] <- pi*SeCu*((1-SeIn)*(1-SeSt)) + (1-pi)*(1-SpCu)*(SpIn*SpSt)

p[2,1,2] <- pi*(1-SeCu)*((1-SeIn)*SeSt) + (1-pi)*SpCu*(SpIn*(1-SpSt))

p[2,2,2] <- pi*(1-SeCu)*((1-SeIn)*(1-SeSt)) + (1-pi)*SpCu*(SpIn*SpSt)

SeIn<-1/(1+exp(-S.SeIn))

S.SeIn ~ dnorm(1.443525,5.09696)

SpIn<-1/(1+exp(-S.SpIn))

S.SpIn ~ dnorm( 2.682732,0.8017619)

SeSt<-1/(1+exp(-S.SeSt))

S.SeSt ~ dnorm(0.8094862,51.55374)##Incorrect prior

SpSt<-1/(1+exp(-S.SpSt))

S.SpSt ~ dnorm( 2.602153, 0.8677139)

SeCu<-1/(1+exp(-S.SeCu))

S.SeCu ~ dnorm(-2.197225,1.364591)

SpCu<-1/(1+exp(-S.SpCu))

S.SpCu ~ dnorm(4.59512, 0.2206248 )

pi<-1/(1+exp(-S.pi))

S.pi ~ dnorm(-1.15268,13.49009)

}

",fill = TRUE)

sink()

# Bundle data

bugs.data <- list(y= my.array,K=2,n=875)

# Initial values

inits <- function(){list(pi=0.24, SeIn=0.81, SpIn=0.94, SeSt=0.49, SpSt=0.93, SeCu=0.10, SpCu=1.00)}

# Parameters monitored

parameters <- c("pi","SeIn","SpIn","SeSt","SpSt","SeCu","SpCu")

# MCMC settings

ni <- 50000

nt <- 10

nb <- 5000

nc <- 3

# Call WinBUGS from R (BRT 1 min)

Inform_incorrect_2 <- bugs(bugs.data, inits=NULL, parameters, "winbugsmodel2.bug", n.chains = nc, n.thin = nt, n.iter = ni, n.burnin = nb, debug = FALSE, bugs.directory = bugs.dir, working.directory = getwd())

print(Inform_incorrect_2,digits=3)

#Plot comparing priors with posteriors

par(mfrow=c(4,2))

plot(density(((Inform_incorrect_2$sims.list$SeSt))),xlim=c(0.01,0.99),lwd=3,main="",bty='n',xlab="Sensitivity Test A")

gaus<-rnorm(10000,0.809486,sqrt(1/51.55374))#0.8094862,51.55374

lines(density(inv.logit(gaus)),xlim=c(0.4,0.75),ylim=c(0,20),col="red",lwd=3,lty=2)

abline(v=0.492,lty=2,lwd=3,col="grey60")

plot(density(((Inform_incorrect_2$sims.list$SpSt))),xlim=c(0.01,0.99),lwd=3,main="",bty='n',xlab="Specificity Test A")

gaus<-rnorm(10000,2.602153,sqrt(1/0.8677139))

lines(density(inv.logit(gaus)),xlim=c(0.4,0.75),ylim=c(0,20),col="red",lwd=3,lty=2)

abline(v=0.931,lty=2,lwd=3,col="grey60")

plot(density(((Inform_incorrect_2$sims.list$SeIn))),xlim=c(0.01,0.99),lwd=3,main="",bty='n',xlab="Sensitivity Test B")

gaus<-rnorm(10000,1.443525,sqrt(1/5.09696))

lines(density(inv.logit(gaus)),xlim=c(0.4,0.75),ylim=c(0,20),col="red",lwd=3,lty=2)

abline(v=0.809,lty=2,lwd=3,col="grey60")

plot(density(((Inform_incorrect_2$sims.list$SpIn))),xlim=c(0.01,0.99),lwd=3,main="",bty='n',xlab="Specificity Test B")

gaus<-rnorm(10000,2.682732,sqrt(1/0.8017619))

lines(density(inv.logit(gaus)),xlim=c(0.4,0.75),ylim=c(0,20),col="red",lwd=3,lty=2)

abline(v=0.936,lty=2,lwd=3,col="grey60")

plot(density(((Inform_incorrect_2$sims.list$SeCu))),xlim=c(0.01,0.99),lwd=3,main="",bty='n',xlab="Sensitivity Test C")

gaus<-rnorm(10000,-2.197225,sqrt(1/1.364591))

lines(density(inv.logit(gaus)),xlim=c(0.4,0.75),ylim=c(0,20),col="red",lwd=3,lty=2)

abline(v=0.1,lty=2,lwd=3,col="grey60")

plot(density(((Inform_incorrect_2$sims.list$SpCu))),xlim=c(0.01,0.99),lwd=3,main="",bty='n',xlab="Specificity Test C")

gaus<-rnorm(10000,4.59512,sqrt(1/0.2206248))

lines(density(inv.logit(gaus)),xlim=c(0.4,0.75),ylim=c(0,20),col="red",lwd=3,lty=2)

abline(v=0.999,lty=2,lwd=3,col="grey60")

plot(density(((Inform_incorrect_2$sims.list$pi))),xlim=c(0.01,0.99),lwd=3,main="",bty='n',xlab="Prevalence")

gaus<-rnorm(10000,-1.15268,sqrt(1/13.49009))

lines(density(inv.logit(gaus)),xlim=c(0.4,0.75),ylim=c(0,20),col="red",lwd=3,lty=2)

abline(v=0.24,lty=2,lwd=3,col="grey60")

#######INFORM -change specificity gamma

###decrease IFNc Sp 0.736 (0.421, 0.787)Drewe et al 2010

mu=logit(0.736)

sigma=(logit(0.736)-logit(0.621))/1.96#as this is 95% CI

1/( sigma^2)#tau= 13.59967

mu# 1.025281

gaus<-rnorm(1000,mu,sigma)

plot(density(inv.logit(gaus)))

sink("winbugsmodel2.bug")

cat("

##model

## Cu=culture, In=IFNg, St=StatPak, Se=sensitivity, Sp=specificity, Pi=prevalence

model {

y[1:K, 1:K, 1:K] ~ dmulti(p[1:K, 1:K, 1:K], n)

p[1,1,1] <- pi*SeCu*(SeIn*SeSt) + (1-pi)*(1-SpCu)*((1-SpIn)*(1-SpSt))

p[1,2,1] <- pi*SeCu*(SeIn*(1-SeSt)) + (1-pi)*(1-SpCu)*((1-SpIn)*SpSt)

p[1,1,2] <- pi*(1-SeCu)*(SeIn*SeSt) + (1-pi)*SpCu*((1-SpIn)*(1-SpSt))

p[1,2,2] <- pi*(1-SeCu)*(SeIn*(1-SeSt)) + (1-pi)*SpCu*((1-SpIn)*SpSt)

p[2,1,1] <- pi*SeCu*((1-SeIn)*SeSt) + (1-pi)*(1-SpCu)*(SpIn*(1-SpSt))

p[2,2,1] <- pi*SeCu*((1-SeIn)*(1-SeSt)) + (1-pi)*(1-SpCu)*(SpIn*SpSt)

p[2,1,2] <- pi*(1-SeCu)*((1-SeIn)*SeSt) + (1-pi)*SpCu*(SpIn*(1-SpSt))

p[2,2,2] <- pi*(1-SeCu)*((1-SeIn)*(1-SeSt)) + (1-pi)*SpCu*(SpIn*SpSt)

SeIn<-1/(1+exp(-S.SeIn))

S.SeIn ~ dnorm(1.443525,5.09696)

SpIn<-1/(1+exp(-S.SpIn))

S.SpIn ~ dnorm(1.025281, 13.59967)###incorrect

SeSt<-1/(1+exp(-S.SeSt))

S.SeSt ~ dnorm(-0.03200273,63.5998)

SpSt<-1/(1+exp(-S.SpSt))

S.SpSt ~ dnorm( 2.602153, 0.8677139)

SeCu<-1/(1+exp(-S.SeCu))

S.SeCu ~ dnorm(-2.197225,1.364591)

SpCu<-1/(1+exp(-S.SpCu))

S.SpCu ~ dnorm(4.59512, 0.2206248 )

pi<-1/(1+exp(-S.pi))

S.pi ~ dnorm(-1.15268,13.49009)

}

",fill = TRUE)

sink()

# Bundle data

bugs.data <- list(y= my.array,K=2,n=875)

# Initial values

inits <- function(){list(pi=0.24, SeIn=0.81, SpIn=0.94, SeSt=0.49, SpSt=0.93, SeCu=0.10, SpCu=1.00)}

# Parameters monitored

parameters <- c("pi","SeIn","SpIn","SeSt","SpSt","SeCu","SpCu")

# MCMC settings

ni <- 50000

nt <- 10

nb <- 5000

nc <- 3

# Call WinBUGS from R (BRT 1 min)

Inform_incorrect_3 <- bugs(bugs.data, inits=NULL, parameters, "winbugsmodel2.bug", n.chains = nc, n.thin = nt, n.iter = ni, n.burnin = nb, debug = FALSE, bugs.directory = bugs.dir, working.directory = getwd())

print(Inform_incorrect_3,digits=3)

#Plot comparing priors with posteriors

par(mfrow=c(4,2))

plot(density(((Inform_incorrect_3$sims.list$SeSt))),xlim=c(0.01,0.99),lwd=3,main="",bty='n',xlab="Sensitivity Test A")

gaus<-rnorm(10000,-0.03200273,sqrt(1/63.5998

))#-0.03200273,63.5998

lines(density(inv.logit(gaus)),xlim=c(0.4,0.75),ylim=c(0,20),col="red",lwd=3,lty=2)

abline(v=0.492,lty=2,lwd=3,col="grey60")

plot(density(((Inform_incorrect_3$sims.list$SpSt))),xlim=c(0.01,0.99),lwd=3,main="",bty='n',xlab="Specificity Test A")

gaus<-rnorm(10000,2.602153,sqrt(1/0.8677139))

lines(density(inv.logit(gaus)),xlim=c(0.4,0.75),ylim=c(0,20),col="red",lwd=3,lty=2)

abline(v=0.931,lty=2,lwd=3,col="grey60")

plot(density(((Inform_incorrect_3$sims.list$SeIn))),xlim=c(0.01,0.99),lwd=3,main="",bty='n',xlab="Sensitivity Test B")

gaus<-rnorm(10000,1.443525,sqrt(1/5.09696))

lines(density(inv.logit(gaus)),xlim=c(0.4,0.75),ylim=c(0,20),col="red",lwd=3,lty=2)

abline(v=0.809,lty=2,lwd=3,col="grey60")

plot(density(((Inform_incorrect_3$sims.list$SpIn))),xlim=c(0.01,0.99),lwd=3,main="",bty='n',xlab="Specificity Test B")

gaus<-rnorm(10000,1.025281,sqrt(1/ 13.59967))#

lines(density(inv.logit(gaus)),xlim=c(0.4,0.75),ylim=c(0,20),col="red",lwd=3,lty=2)

abline(v=0.936,lty=2,lwd=3,col="grey60")

plot(density(((Inform_incorrect_3$sims.list$SeCu))),xlim=c(0.01,0.99),lwd=3,main="",bty='n',xlab="Sensitivity Test C")

gaus<-rnorm(10000,-2.197225,sqrt(1/1.364591))

lines(density(inv.logit(gaus)),xlim=c(0.4,0.75),ylim=c(0,20),col="red",lwd=3,lty=2)

abline(v=0.1,lty=2,lwd=3,col="grey60")

plot(density(((Inform_incorrect_3$sims.list$SpCu))),xlim=c(0.01,0.99),lwd=3,main="",bty='n',xlab="Specificity Test C")

gaus<-rnorm(10000,4.59512,sqrt(1/0.2206248))

lines(density(inv.logit(gaus)),xlim=c(0.4,0.75),ylim=c(0,20),col="red",lwd=3,lty=2)

abline(v=0.999,lty=2,lwd=3,col="grey60")

plot(density(((Inform_incorrect_3$sims.list$pi))),xlim=c(0.01,0.99),lwd=3,main="",bty='n',xlab="Prevalence")

gaus<-rnorm(10000,-1.15268,sqrt(1/13.49009))

lines(density(inv.logit(gaus)),xlim=c(0.4,0.75),ylim=c(0,20),col="red",lwd=3,lty=2)

abline(v=0.24,lty=2,lwd=3,col="grey60")

#######INFORM -change specificity gamma

###decrease IFNc Sp 0.99 (0.421, 0.787)Drewe et al 2010

mu=logit(0.999)

sigma=(logit(0.999)-logit(0.990))/1.96#as this is 95% CI

1/( sigma^2)#tau= 0.7189085

mu# 6.906755

gaus<-rnorm(1000,mu,sigma)

plot(density(inv.logit(gaus)))

sink("winbugsmodel2.bug")

cat("

##model

## Cu=culture, In=IFNg, St=StatPak, Se=sensitivity, Sp=specificity, Pi=prevalence

model {

y[1:K, 1:K, 1:K] ~ dmulti(p[1:K, 1:K, 1:K], n)

p[1,1,1] <- pi*SeCu*(SeIn*SeSt) + (1-pi)*(1-SpCu)*((1-SpIn)*(1-SpSt))

p[1,2,1] <- pi*SeCu*(SeIn*(1-SeSt)) + (1-pi)*(1-SpCu)*((1-SpIn)*SpSt)

p[1,1,2] <- pi*(1-SeCu)*(SeIn*SeSt) + (1-pi)*SpCu*((1-SpIn)*(1-SpSt))

p[1,2,2] <- pi*(1-SeCu)*(SeIn*(1-SeSt)) + (1-pi)*SpCu*((1-SpIn)*SpSt)

p[2,1,1] <- pi*SeCu*((1-SeIn)*SeSt) + (1-pi)*(1-SpCu)*(SpIn*(1-SpSt))

p[2,2,1] <- pi*SeCu*((1-SeIn)*(1-SeSt)) + (1-pi)*(1-SpCu)*(SpIn*SpSt)

p[2,1,2] <- pi*(1-SeCu)*((1-SeIn)*SeSt) + (1-pi)*SpCu*(SpIn*(1-SpSt))

p[2,2,2] <- pi*(1-SeCu)*((1-SeIn)*(1-SeSt)) + (1-pi)*SpCu*(SpIn*SpSt)

SeIn<-1/(1+exp(-S.SeIn))

S.SeIn ~ dnorm(1.443525,5.09696)

SpIn<-1/(1+exp(-S.SpIn))

S.SpIn ~ dnorm( 6.906755, 0.7189085)###incorrect

SeSt<-1/(1+exp(-S.SeSt))

S.SeSt ~ dnorm(-0.03200273,63.5998)

SpSt<-1/(1+exp(-S.SpSt))

S.SpSt ~ dnorm( 2.602153, 0.8677139)

SeCu<-1/(1+exp(-S.SeCu))

S.SeCu ~ dnorm(-2.197225,1.364591)

SpCu<-1/(1+exp(-S.SpCu))

S.SpCu ~ dnorm(4.59512, 0.2206248 )

pi<-1/(1+exp(-S.pi))

S.pi ~ dnorm(-1.15268,13.49009)

}

",fill = TRUE)

sink()

# Bundle data

bugs.data <- list(y= my.array,K=2,n=875)

# Initial values

inits <- function(){list(pi=0.24, SeIn=0.81, SpIn=0.94, SeSt=0.49, SpSt=0.93, SeCu=0.10, SpCu=1.00)}

# Parameters monitored

parameters <- c("pi","SeIn","SpIn","SeSt","SpSt","SeCu","SpCu")

# MCMC settings

ni <- 50000

nt <- 10

nb <- 5000

nc <- 3

# Call WinBUGS from R (BRT 1 min)

Inform_incorrect_4 <- bugs(bugs.data, inits=NULL, parameters, "winbugsmodel2.bug", n.chains = nc, n.thin = nt, n.iter = ni, n.burnin = nb, debug = FALSE, bugs.directory = bugs.dir, working.directory = getwd())

print(Inform_incorrect_4,digits=3)

#Plot comparing priors with posteriors

par(mfrow=c(4,2))

plot(density(((Inform_incorrect_4$sims.list$SeSt))),xlim=c(0.01,0.99),lwd=3,main="",bty='n',xlab="Sensitivity Test A")

gaus<-rnorm(10000,-0.03200273,sqrt(1/63.5998

))#-0.03200273,63.5998

lines(density(inv.logit(gaus)),xlim=c(0.4,0.75),ylim=c(0,20),col="red",lwd=3,lty=2)

abline(v=0.492,lty=2,lwd=3,col="grey60")

plot(density(((Inform_incorrect_4$sims.list$SpSt))),xlim=c(0.01,0.99),lwd=3,main="",bty='n',xlab="Specificity Test A")

gaus<-rnorm(10000,2.602153,sqrt(1/0.8677139))

lines(density(inv.logit(gaus)),xlim=c(0.4,0.75),ylim=c(0,20),col="red",lwd=3,lty=2)

abline(v=0.931,lty=2,lwd=3,col="grey60")

plot(density(((Inform_incorrect_4$sims.list$SeIn))),xlim=c(0.01,0.99),lwd=3,main="",bty='n',xlab="Sensitivity Test B")

gaus<-rnorm(10000,1.443525,sqrt(1/5.09696))

lines(density(inv.logit(gaus)),xlim=c(0.4,0.75),ylim=c(0,20),col="red",lwd=3,lty=2)

abline(v=0.809,lty=2,lwd=3,col="grey60")

plot(density(((Inform_incorrect_4$sims.list$SpIn))),xlim=c(0.01,0.99),lwd=3,main="",bty='n',xlab="Specificity Test B")

gaus<-rnorm(10000, 6.906755,sqrt(1/ 0.7189085))#

lines(density(inv.logit(gaus)),xlim=c(0.4,0.75),ylim=c(0,20),col="red",lwd=3,lty=2)

abline(v=0.936,lty=2,lwd=3,col="grey60")

plot(density(((Inform_incorrect_4$sims.list$SeCu))),xlim=c(0.01,0.99),lwd=3,main="",bty='n',xlab="Sensitivity Test C")

gaus<-rnorm(10000,-2.197225,sqrt(1/1.364591))

lines(density(inv.logit(gaus)),xlim=c(0.4,0.75),ylim=c(0,20),col="red",lwd=3,lty=2)

abline(v=0.1,lty=2,lwd=3,col="grey60")

plot(density(((Inform_incorrect_4$sims.list$SpCu))),xlim=c(0.01,0.99),lwd=3,main="",bty='n',xlab="Specificity Test C")

gaus<-rnorm(10000,4.59512,sqrt(1/0.2206248))

lines(density(inv.logit(gaus)),xlim=c(0.4,0.75),ylim=c(0,20),col="red",lwd=3,lty=2)

abline(v=0.999,lty=2,lwd=3,col="grey60")

plot(density(((Inform_incorrect_4$sims.list$pi))),xlim=c(0.01,0.99),lwd=3,main="",bty='n',xlab="Prevalence")

gaus<-rnorm(10000,-1.15268,sqrt(1/13.49009))

lines(density(inv.logit(gaus)),xlim=c(0.4,0.75),ylim=c(0,20),col="red",lwd=3,lty=2)

abline(v=0.24,lty=2,lwd=3,col="grey60")

##############################################################

#Vague priors

#######UNINFORM

sink("constant_uninform.bug")

cat("

##model

## Cu=culture, In=IFNg, St=StatPak, Se=sensitivity, Sp=specificity, Pi=prevalence

model {

y[1:K, 1:K, 1:K] ~ dmulti(p[1:K, 1:K, 1:K], n)

p[1,1,1] <- (pi*SeCu*SeIn*SeSt) + ((1-pi)*(1-SpCu)*(1-SpIn)*(1-SpSt))#

p[1,2,1] <- (pi*SeCu*SeIn*(1-SeSt)) + ((1-pi)*(1-SpCu)*(1-SpIn)*SpSt)#

p[1,1,2] <- (pi*(1-SeCu)*SeIn*SeSt) + ((1-pi)*SpCu*(1-SpIn)*(1-SpSt))#

p[1,2,2] <- (pi*(1-SeCu)*SeIn*(1-SeSt)) + ((1-pi)*SpCu*(1-SpIn)*SpSt) #

p[2,1,1] <- (pi*SeCu*(1-SeIn)*SeSt) + ((1-pi)*(1-SpCu)*SpIn*(1-SpSt))#

p[2,2,1] <- (pi*SeCu*(1-SeIn)*(1-SeSt)) + ((1-pi)*(1-SpCu)*SpIn*SpSt) #

p[2,1,2] <- (pi*(1-SeCu)*(1-SeIn)*SeSt) + ((1-pi)*SpCu*SpIn*(1-SpSt)) #

p[2,2,2] <- (pi*(1-SeCu)*(1-SeIn)*(1-SeSt)) + ((1-pi)*SpCu*SpIn*SpSt)

SeIn<-1/(1+exp(-S.SeIn))

S.SeIn~ dnorm(0,0.368)

SpIn<-1/(1+exp(-S.SpIn))

S.SpIn~ dnorm(0,0.368)

SeSt<-1/(1+exp(-S.SeSt))

S.SeSt ~ dnorm(0,0.368)

SpSt<-1/(1+exp(-S.SpSt))

S.SpSt ~ dnorm(0,0.368)

SeCu<-1/(1+exp(-S.SeCu))

S.SeCu ~ dnorm(0,0.368)

SpCu<-1/(1+exp(-S.SpCu))

S.SpCu ~ dnorm(0,0.368)

pi<-1/(1+exp(-S.pi))

S.pi~ dnorm(0,0.368)

}

",fill = TRUE)

sink()

sum(my.array)

# Bundle data

bugs.data <- list(y= my.array,K=2,n=875)

# Initial values

inits <- function(){list(S.SeIn =0.443525,S.SpIn=2.682732,S.SeSt=-0.03200273,S.SpSt= 2.602153,

S.SeCu=-2.197225,S.SpCu=4.59512,S.pi=-1.152680)}

# Parameters monitored

parameters <- c("pi","SeIn","SpIn","SeSt","SpSt","SeCu","SpCu")

# MCMC settings

ni <- 50000

nt <- 10

nb <- 5000

nc <- 3

# Call WinBUGS from R (BRT 1 min)

Uninform<- bugs(bugs.data, inits, parameters, "constant_uninform.bug", n.chains = nc, n.thin = nt, n.iter = ni, n.burnin = nb, debug = FALSE, bugs.directory = bugs.dir, working.directory = getwd())

print(Uninform,digits=3)

###plot comparing priors and posteriors

pdf("Fig 2 vague priors.pdf")

par(mfrow=c(4,2),mar=c(5,4,1,1)+0.1,cex.lab=1.2)

gaus<-rnorm(10000,0,sqrt(1/0.368))

plot(density(((Uninform$sims.list$SeSt))),xlim=c(0.01,0.99),lwd=2,main="",bty='n',xlab="Sensitivity Test A")

lines(density(inv.logit(gaus)),xlim=c(0.4,0.75),ylim=c(0,20),col="red",lwd=3,lty=2)

abline(v=0.492,lty=2,lwd=3,col="grey60")

plot(density(((Uninform$sims.list$SpSt))),xlim=c(0.01,0.99),lwd=2,main="",bty='n',xlab="Specificity Test A")

lines(density(inv.logit(gaus)),xlim=c(0.4,0.75),ylim=c(0,20),col="red",lwd=3,lty=2)

abline(v=0.931,lty=2,lwd=3,col="grey60")

plot(density(((Uninform$sims.list$SeIn))),xlim=c(0.01,0.99),lwd=2,main="",bty='n',xlab="Sensitivity Test B")

lines(density(inv.logit(gaus)),xlim=c(0.4,0.75),ylim=c(0,20),col="red",lwd=3,lty=2)

abline(v=0.809,lty=2,lwd=3,col="grey60")

plot(density(((Uninform$sims.list$SpIn))),xlim=c(0.01,0.99),lwd=2,main="",bty='n',xlab="Specificity Test B")

lines(density(inv.logit(gaus)),xlim=c(0.4,0.75),ylim=c(0,20),col="red",lwd=3,lty=2)

abline(v=0.936,lty=2,lwd=3,col="grey60")

plot(density(((Uninform$sims.list$SeCu))),xlim=c(0.01,0.99),lwd=2,main="",bty='n',xlab="Sensitivity Test C")

lines(density(inv.logit(gaus)),xlim=c(0.4,0.75),ylim=c(0,20),col="red",lwd=3,lty=2)

abline(v=0.1,lty=2,lwd=3,col="grey60")

plot(density(((Uninform$sims.list$SpCu))),xlim=c(0.01,0.99),lwd=2,main="",bty='n',xlab="Specificity Test C")

lines(density(inv.logit(gaus)),xlim=c(0.4,0.75),ylim=c(0,20),col="red",lwd=3,lty=2)

abline(v=0.999,lty=2,lwd=3,col="grey60")

plot(density(((Uninform$sims.list$pi))),xlim=c(0.01,0.99),lwd=2,main="",bty='n',xlab="Prevalence")

lines(density(inv.logit(gaus)),xlim=c(0.4,0.75),ylim=c(0,20),col="red",lwd=3,lty=2)

abline(v=0.24,lty=2,lwd=3,col="grey60")

#vioplot(inv.logit(gaus),Uninform$sims.list$SeSt,)

#abline(h=0.24,lty=2,lwd=3,col="grey60")

dev.off()

###comparison of prevalence

library(vioplot)

pdf("Fig3 posteriors.pdf")

opar<-par(mar=c(8,4,3,1)+0.1,las=3)

d<-vioplot(Inform_incorrect_1$sims.list$pi,Inform_incorrect_2$sims.list$pi,Inform_incorrect_3$sims.list$pi,Inform_incorrect_4$sims.list$pi,Uninform$sims.list$pi,

Inform$sims.list$pi,drawRect=FALSE,ylim=c(0,.5),names=c("Sens A Low","Sens A High","Spec B Low","Spec B High","Imprecise Priors","Precise Priors"))

mtext("Prevalence",side=2,line=3)

abline(h=0.24,lty=2,lwd=3,col="grey60")

par(opar)

#print(Inform_incorrect_1)

points(1,Inform_incorrect_1$summary[1,1],pch=16,cex=2)

arrows(1,Inform_incorrect_1$summary[1,3],1,Inform_incorrect_1$summary[1,7],code=3,angle=90,length=0.1)

points(2,Inform_incorrect_2$summary[1,1],pch=16,cex=2)

arrows(2,Inform_incorrect_2$summary[1,3],2,Inform_incorrect_2$summary[1,7],code=3,angle=90,length=0.1)

points(3,Inform_incorrect_3$summary[1,1],pch=16,cex=2)

arrows(3,Inform_incorrect_3$summary[1,3],3,Inform_incorrect_3$summary[1,7],code=3,angle=90,length=0.1)

points(4,Inform_incorrect_4$summary[1,1],pch=16,cex=2)

arrows(4,Inform_incorrect_4$summary[1,3],4,Inform_incorrect_4$summary[1,7],code=3,angle=90,length=0.1)

points(5,Uninform$summary[1,1],pch=16,cex=2)

arrows(5,Uninform$summary[1,3],5,Uninform$summary[1,7],code=3,angle=90,length=0.1)

points(6,Inform$summary[1,1],pch=16,cex=2)

arrows(6,Inform$summary[1,3],6,Inform$summary[1,7],code=3,angle=90,length=0.1)

dev.off()
